# Modulation of the NF-κB Pathway by *Bordetella pertussis* Filamentous Hemagglutinin

**DOI:** 10.1371/journal.pone.0003825

**Published:** 2008-11-27

**Authors:** Tzvia Abramson, Hassya Kedem, David A. Relman

**Affiliations:** 1 Department of Microbiology and Immunology, Stanford University School of Medicine, Stanford, California, United States of America; 2 Department of Medicine, Stanford University School of Medicine, Stanford, California, United States of America; 3 Veterans Affairs Palo Alto Health Care System, Palo Alto, California, United States of America; 4 Department of Biological Sciences, San Jose State University, San Jose, California, United States of America; Columbia University, United States of America

## Abstract

**Background:**

Filamentous hemagglutinin (FHA) is a cell-associated and secreted adhesin produced by *Bordetella pertussis* with pro-apoptotic and pro-inflammatory activity in host cells. Given the importance of the NF-κB transcription factor family in these host cell responses, we examined the effect of FHA on NF-κB activation in macrophages and bronchial epithelial cells, both of which are relevant cell types during natural infection.

**Methodology/Principal Findings:**

Exposure to FHA of primary human monocytes and transformed U-937 macrophages, but not BEAS-2B epithelial cells, resulted in early activation of the NF-κB pathway, as manifested by the degradation of cytosolic IκBα, by NF-κB DNA binding, and by the subsequent secretion of NF-κB-regulated inflammatory cytokines. However, exposure of macrophages and human monocytes to FHA for two hours or more resulted in the accumulation of cytosolic IκBα, and the failure of TNF-α to activate NF-κB. Proteasome activity was attenuated following exposure of cells to FHA for 2 hours, as was the nuclear translocation of RelA in BEAS-2B cells.

**Conclusions:**

These results reveal a complex temporal dynamic, and suggest that despite short term effects to the contrary, longer exposures of host cells to this secreted adhesin may block NF-κB activation, and perhaps lead to a compromised immune response to this bacterial pathogen.

## Introduction

Despite widespread vaccination against *Bordetella pertussis (B. pertussis)*, whooping cough is reemerging in most parts of the world. *B. pertussis* colonizes the mucosa of the respiratory tract, where it interacts with ciliated bronchial epithelial cells and local immune cells, causing a highly contagious and prolonged respiratory disease [1]. Filamentous hemagglutinin (FHA) is a 220 kDa protein associated with the bacterial surface, but also secreted in substantial amounts [2]. Studies using FHA-deficient *B. pertussis* strains implicate this protein in tracheal colonization, cell adherence [3] and invasion [4] of macrophages and epithelial cells by *B. pertussis*. Galactose-dependent lectins and integrin receptors such as α_M_β_2_ and α_v_β_3_ have been suggested as eukaryotic binding sites for FHA [5], [6].

*In vitro* and *in vivo* studies indicate that soluble FHA triggers several immunomodulatory responses, as manifested by the secretion of both inflammatory and anti-inflammatory cytokines by macrophages [7], [8], as well as by the induction of FHA-specific T regulatory cells in a respiratory model [9].

NF-κB transcription factors mediate a large number of innate immunomodulatory responses [10]. A number of pathogens have evolved to manipulate this system in order to enhance their survival in the host [11]. With the goal of identifying the mechanisms by which FHA contributes to persistent and prolonged *B. pertussis* infections, we analyzed the interaction of FHA with components of the NF-κB pathway.

The NF-κB transcription factor pathway is activated by a variety of microbial components that signal through innate immune toll-like-receptors (TLR) and initiate the transcription of genes associated with a spectrum of inflammatory responses [12]. NF-κB is a family of transcription-factor proteins; RelA/p50 is the most well-studied member. This heterodimer is sequestered in the cytoplasm of resting cells by the inhibitory protein IκBα. Activation of the classical NF-κB pathway follows from IKKβ kinase-dependent serine phosphorylation of IκBα followed by IκBα ubiquitination. This modification marks IκBα and subjects it to rapid proteasomal degradation, resulting in the release of RelA/p50 heterodimers and their translocation to the nucleus. RelA/p50 then binds to κB DNA sites and initiates gene transcription [13]. Inhibition of the NF-κB pathway has been linked to attenuated inflammatory activity and apoptosis [14].

In the current study, we examined the ability of *B. pertussis* FHA to affect activation of the NF-κB pathway as a possible strategy by this pathogen to attenuate the immune response and prolong its survival in the host.

## Materials and Methods

### Eukaryotic cell lines and primary cell culture

The human monocyte-like cell line, U-937, and the human bronchial epithelial cell line, BEAS-2B, were obtained from the American Type Culture Collection (ATCC) (Rockville, MD) (#CRL-15932 and #CRL-9606, respectively) and maintained in culture using recommended conditions. U-937 cells were induced to differentiate into macrophage-like cells by treatment with 10 ng/ml of phorbol 12-myristate 13-acetate (PMA) (Sigma, St. Louis, MO), as previously described [8]. A-431 (epidermoid carcinoma cell) extracts were used as a positive control in IκBα immunoblots (Santa Cruz Biotechnology, Santa Cruz, CA). Fresh human peripheral blood monocytes were obtained from healthy donors. The use of these subjects was approved by the Stanford University Administrative Panel on Human Subjects in Medical Research. PBMCs (peripheral blood mononuclear cells) were isolated from a Ficoll gradient. Monocytes were further purified with the Miltenyi Biotec MACS magnetic beads separation technology (Auburn, CA). Monocytes were placed in tissue culture plates one day prior to the experiment. Non-adherent cells were removed just prior to the experiment.

### Reagents and antibodies

Purified *B. pertussis* FHA was kindly provided by Rino Rappuoli and Mariagrazia Pizza (Novartis Vaccines, Siena, Italy) at a concentration of 1 mg/ml. At Novartis Vaccines, FHA was purified from culture supernatant of *Bordetella pertussis* Tohama I using anion exchange chromatography (Matrex Cellufine Sulfate, Millipore Corp., Watford, United Kindom), and filtration with Millipack 100 filters. Enzyme linked immunosorbent assay (ELISA) did not reveal the presence of pertactin, adenylate cyclase toxin, or pertussis toxin in these FHA preparations. We estimated endotoxin content using the Limulus amoebocyte lysate assay (QCL-1000kit; BioWhittaker, Walkersville, MD), and found that the FHA preparation used in these experiments contained 2 EU of endotoxin units per microgram FHA protein, or 10 EU per ml under assay conditions. We used *B. pertussis* LPS (List Biological, Campbell, CA) as a control for this assay. In a previous publication [8], we showed that FHA induces far more TNF-α secretion by U-937 cells than would be expected from the known amounts of LPS that contaminate these FHA preparations, based on a comparison of the endotoxin activity in FHA and LPS preparations needed to induce secretion of equivalent levels of TNF-α.

Human TNF-α was purchased from R&D Systems (Minneapolis, MN). Anti-RelA (C-20) and anti-C-terminal IκBα (C-21) antibodies were purchased from Santa Cruz Biotechnology. Alexa-fluor 568 goat anti-rabbit antibody was purchased from Molecular Probes (Eugene, OR), and anti-actin AC-40 antibody was purchased from Sigma.

### ELISA

IL-8, IL-10 and TNF-α were measured in cell tissue culture supernatants with solid phase sandwich ELISA kits (BioSource International, Camarillo, CA) with the following lower limits of detection: 0.39, 7.8, and 1 pg/ml, respectively.

### Western blot analysis

Whole-cell lysates were prepared and immunoblots performed as previously described [8].

### Electrophoretic mobility shift assay (EMSA)

To prepare nuclear and cytosolic extracts, 10^7^ cells were harvested and washed with ice-cold PBS, centrifuged at 250×g and re-suspended in 150 µl of cold buffer A (10 mM Tris–HCl 7.4, 10 mM NaCl, 3 mM MgCl_2_, 100 µg/ml PMSF, 0.5 mM DTT and 1 mM Na_3_O_4_V). Cells were allowed to swell on ice for 30 minutes, and then 10 µl of 10% NP-40 was added. Cells were mixed vigorously for 20 seconds and centrifuged for 10 seconds at 16,000×g. The supernatant, containing the cytoplasmic fraction, was centrifuged again for 10 minutes at 16,000×g. The protein content of the supernatant was determined with the BCA protein assay (Pierce, Rockford, IL). The supernatant was mixed with 6x loading buffer (denatured) and stored at −20°C for immunoblot analysis. The nuclei were rinsed with buffer A, and centrifuged for 10 seconds at 16,000×g. The pellet was re-suspended in 60 µl buffer C (20 mm HEPES pH 7.9, 420 mM NaCl, 1.5 mM MgCl_2_, 0.2 mM EDTA, 25% glycerol, 100 µg/ml PMSF, 0.5 mM DTT, 100 µM Na_3_O_4_V, 3 µg/ml aprotinin, and 1 µg/ml leupeptine (Roche, Indianapolis, IN) for 40 minutes on ice. After five minutes centrifugation at 16,000×g, the supernatant containing the nuclear fraction was collected, aliquoted and stored at −80°C until its analysis by EMSA.

### EMSA analysis

Oligonucleotide probes containing NF-κB enhancer sequences, as well as the rest of the assay components (“Gel shift assay core system”), were purchased from Promega (Madison, WI) and used according to the manufacturer's instructions. Briefly, DNA protein-binding reactions were performed for 10 minutes at room temperature with 15 µg nuclear extract, 1 µl P^32^ labeled oligo, and 4 µl of binding buffer containing poly dI/dC. Samples were then separated by electrophoresis in a 4% non-denaturing acrylamide gel containing 0.5X TRIS-borate EDTA. Gels were dried and analyzed by phosphorimaging (Molecular Dynamics, Sunnyvale, CA).

### Proteasome activity

Cells were lysed in 0.3 ml proteasome lysing buffer (50 mM TRIS-HCl pH 8, 140 mM KCl, 10 mM glucose, 2 mM ATP, 5 mM MgCl_2_ and 1 mM EDTA), with the following protease inhibitors added just before use: 5 mM dithiothreitol, 0.1 mM phenymethylsulfonyl fluoride, 2.2 µg/1 ml aprotonin and 2 µg/ml leupeptin. The pellet was sonicated for 30 seconds and incubated on ice for 30 minutes. The cell lysate was centrifuged for 10 minutes at 14,000 rpm at 4°C. The supernatant (cytosolic extract) was stored at −80°C until the enzymatic assay was performed.

Cytosolic extract (5 ug of total protein) was incubated in a total of 100 µl of assay buffer containing 1M HEPES, 0.5 mM EDTA, 0.035% SDS and 70 mM of the substrate Suc-LLVY-AMC for 15 minutes at room temperature. Accumulation of the fluorophore AMC was measured in a 96-well plate fluorometer (Gemini II, Molecular Device) at an excitation wavelength of 360 nm and emission wavelength of 460 nm, every 15 minutes for 45 minutes. Activity was estimated by subtracting the fluorescence obtained for proteasome-independent activity (in the presence of 60 mM MG-132 proteasome inhibitor) from the values obtained in its absence. The values shown represent the ratio of the proteasome activity in each sample to that of the basal activity of the untreated extracts.

## Results

### FHA induces rapid degradation of IκBα in macrophages but not in epithelial cells

**Figure 1:**
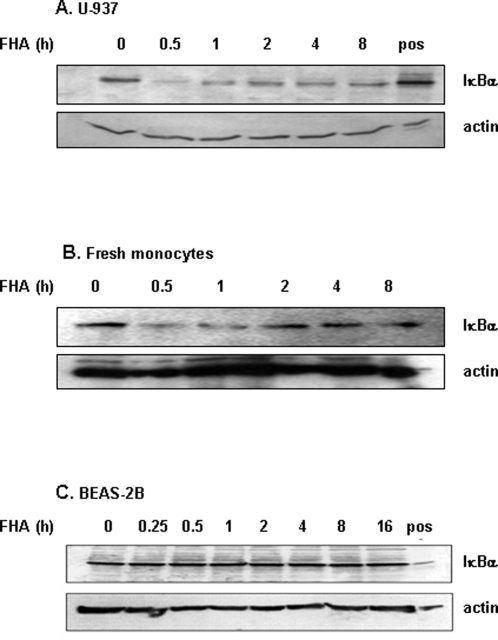
IκBα cytosolic levels in FHA-treated U-937 monocyte-derived macrophages, fresh human monocytes and BEAS-2B bronchial epithelial cells. (A) U-937 derived macrophages, (B) fresh human monocytes and (C) BEAS-2B cells were treated with 5 µg/ml FHA for the indicated time periods. Cytoplasmic protein extracts were analyzed by immunoblot procedures with an antibody directed against the C-terminus of IκBα. A-431 cell lysate was used as a positive control to identify IκBα. Equal amounts of loaded protein were confirmed by anti-actin immunoblotting. This is a representative experiment of four performed under similar conditions.

We analyzed the effect of FHA on the NF-κB transcription-factor pathway by incubating U-937 monocyte-derived macrophages, fresh human monocytes or the human bronchial epithelial cell line BEAS-2B with 5 µg/ml FHA for up to 16 hours (Fig. 1A, 1B 1C). NF-κB activation was assessed by measuring the cytosolic levels of IκBα inhibitory protein. Proteasomal degradation of IκBα is a key step that precedes nuclear translocation of RelA/p50 in activated cells. Both monocyte-derived cells (Figs. 1A, 1B) and epithelial cells (Fig. 1C) express significant levels of IκBα under resting conditions. IκBα cytosolic levels in monocyte-derived cells treated for various periods of time (0–8 hours) with 5 µg/ml FHA suggested rapid degradation of IκBα, observable within 30 minutes in U-937 macrophages (Fig. 1A) and in fresh monocytes (Fig. 1B). The levels of IκBα returned to baseline within two hours in both cell types, and no further degradation was observed. However, in BEAS-2B bronchial epithelial cells, IκBα was not degraded upon similar treatment with FHA (Fig. 1C). The putative IκBα band was confirmed as such using lysates from A-431 cells, which over-express this molecule. These data suggest that FHA is capable of inducing a rapid activation of the NF-κB transcription factor pathway in monocyte-derived cells, but does not appear to do so in epithelial cells.

### NF-κB–DNA binding activity in FHA-treated cells

**Figure 2:**
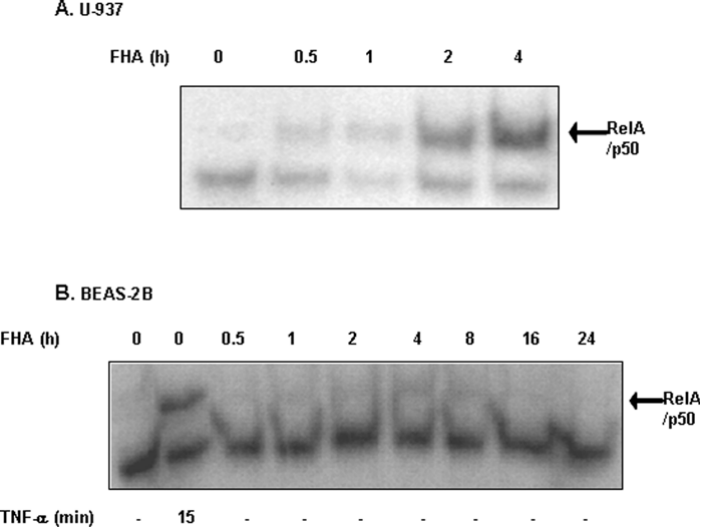
NF-κB–DNA binding activity in U-937 monocyte-derived macrophages and BEAS-2B bronchial epithelial cells treated with FHA. Nuclear extracts from U-937 derived macrophages (A) and BEAS-2B cells (B) were treated as indicated and analyzed for DNA binding activity to κB responsive elements by EMSA. The migration position of the NF-κB heterodimeric-binding complex is indicated, as determined previously in TNF-α treated cells, using super-shift analysis with unlabeled NF-κB oligo and anti-p50 (Fig. S1). These are representative experiments of three sets performed under similar conditions.

To assess NF-κB pathway activation further, we measured NF-κB–DNA binding in the nuclear fractions of FHA-treated cells. U-937 cells treated with 5 µg/ml FHA for up to four hours revealed increased binding of RelA/p50 to κB–DNA binding sites in a time-dependent manner, with significant levels of increased binding at two and four hours (Fig. 2A). The specificity of binding, as well as the nature of the transcription factors were confirmed by displacement analyses with unlabeled NF-κB oligo and a supershift analysis with p50 antibody (Fig. S1). Consistent with the results shown in Fig. 1, FHA did not induce NF-κB–DNA binding in BEAS-2B cells. However, TNF-α, a classical activator of NF-κB, induced DNA binding within 15 minutes, confirming the ability of the BEAS-2B cells to activate this transcription factor in response to signals other than FHA (Fig. 2B).

### FHA induces both inflammatory and anti-inflammatory cytokines in monocyte- derived cells but not in a bronchial epithelial cell line

The NF-κB pathway is responsible for the transcriptional activation of genes encoding a variety of inflammatory cytokines. We found that macrophage-like transformed cells and primary monocytes exposed to 5 µg/ml FHA for two hours secreted significantly higher levels of TNF-α and IL-8 than did untreated cells, as detected by ELISA. TNF-α and IL-8 levels from FHA-treated U-937 macrophages were 14- and 12-fold higher than those from untreated cells (Table 1). TNF-α and IL-8 levels from fresh monocytes reached 4.24±0.04 ng/ml and 76±2.3 ng/ml, respectively, while in untreated cells these cytokines remained below the limits of detection (1.0 and 0.39 pg/ml, respectively). No TNF-α was detected in the supernatant of FHA-treated BEAS -2B cells, but 1.34±0.069 ng/ml of IL-8 was detected following treatment (12 fold increase, Table 1), indicating perhaps a role for a transcription factor other than NF-κB. Concomitant with the FHA induction of inflammatory cytokines in monocyte-derived cells, the anti-inflammatory cytokine IL-10 was also produced: U-937 macrophages, 2.2±0.071 ng/ml, 170 fold increase, and primary monocytes, 0.47±0.1 ng/ml. No IL-10 was detected in BEAS-2B cells treated with FHA. These data support the hypothesis that FHA can induce NF-κB activity, but suggest a complex set of downstream effects.

**Table 1:** FHA-induced cytokine secretion in U-937 derived macrophages, fresh human monocytes, and BEAS-2B cells.

		TNF-α (ng/ml)	IL-8 (ng/ml)	IL-10 (ng/ml)
U-937 cells	media only	0.025±0.015	2.7±0.3	0.013±0.001
	+ FHA	0.360±0.02	32±3.2	2.2±0.071
Fresh monocytes	media only	ND	ND	ND
	+ FHA	4.24±0.04	76±2.3	0.47±0.1
BEAS-2B cells	media only	ND	0.108±0.008	ND
	+ FHA	ND	1.341±0.069	ND

Cells were incubated for two hours with 5 µg/ml FHA, washed and further incubated for 16 hours with media alone. TNF-α, IL-8, and IL-10 levels were analyzed in cell supernatants by ELISA. These are representative values from one of three experiments performed under similar conditions. Statistical significance was analyzed by t test (two-sample test, assuming equal variances); FHA induced significant responses in all cases where cytokines could be detected (p < 0.000001).

ND = not detected.

### Prolonged exposure to FHA inhibits proteasomal activity and prevents activation of the NF-κB pathway

**Figure 3:**
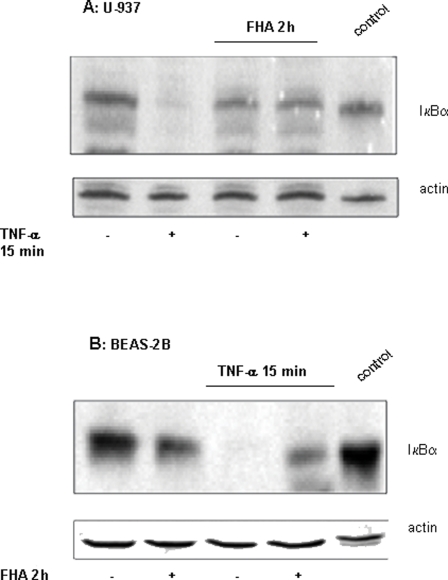
IκBα cytosolic levels in U-937 monocyte-derived macrophages and BEAS-2B bronchial epithelial cells treated with FHA, TNF-α, or both. U-937 derived macrophages (A) and BEAS-2B cells (B) were treated for the indicated time periods with 10 ng/ml TNF-α or 5 µg/ml FHA, or both. Cytoplasmic protein extracts were analyzed by immunoblot procedures with an antibody directed against the C-terminus of IκBα. A-431 cell lysate was used as a positive control to identify IκBα. Equal amounts of loaded protein were confirmed by anti-actin immunoblotting. This is a representative experiment of four performed under similar conditions.

Since immune and other host cells may be exposed to FHA over extended periods of time during the course of infection, we were interested in the cellular responses triggered by more prolonged incubation with FHA. Activation of the NF-κB pathway by a variety of signals results in cyclical fluctuation of IκBα levels in the cytosol [15]. IκBα is constantly degraded in response to a positive signal, but is quickly synthesized in the nucleus and transported back to the cytosol such that the original levels are restored. This pattern is repeated as long as the cells are exposed to a positive signal. However, the activation of the NF-κB pathway by FHA resulted in a different response. Treatment for 15 minutes with 10 ng/ml TNF-α induced complete degradation of IκBα in U-937 macrophages (Fig. 3A), as well as in fresh monocytes (data not shown) and in BEAS-2B bronchial epithelial cells (Fig. 3B). This degradation appeared to be proteasomally mediated, based on the use of the proteasome inhibitor ALLN208719 (Fig. S2). Surprisingly, two hours of pretreatment with 5 µg/ml FHA completely prevented the degradation of IκBα induced by TNF-α in U-937 cells (Fig. 3A) and partially prevented the degradation in BEAS-2B cells (Fig. 3B). These data suggest that prolonged exposure of cells to FHA does not further activate the NF-κB pathway; moreover, such exposure appears to prevent further activation by other stimuli, such as TNF-α. Similar results demonstrating FHA inhibition of IκBα degradation were obtained with Bp-LPS and IL-1β (Figs. S2, S3).

### FHA inhibits the induced nuclear translocation of RelA in BEAS-2B cells

**Figure 4:**
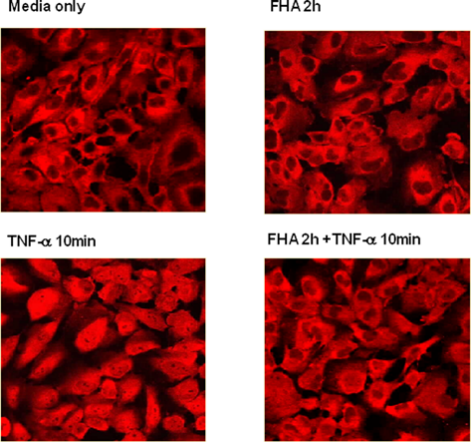
Nuclear translocation of the RelA NF-κB subunit in BEAS-2B epithelial cells. BEAS-2B cells were incubated with either media alone (A), 5 µg/ml FHA for two hours (B), 10 ng/ml TNF-α for 15 minutes (C), or 5 µg/ml FHA for two hours and then 10 ng/ml TNF-α for 15 minutes (D). Cells were fixed, permeabilized and labeled with an antibody specific for the NF-κB RelA subunit, followed by an Alexa 568-labeled secondary antibody, and observed by confocal microscopy, as described in Materials and Methods. This is a representative experiment of three performed under similar conditions.

Activation of the NF-κB pathway results in the dissociation of NF-κB molecules from IκBα and their translocation to the nucleus. We took advantage of the flat morphology of BEAS-2B cells and their large cytoplasmic areas to analyze, by immunofluorescent staining, the translocation of RelA from cytosol to nucleus. Following treatment with FHA, TNF-α, or both, cells were stained with a fluorescent antibody for RelA, as described in Methods. Untreated and two-hour FHA-treated cells revealed that RelA was localized primarily in the cytosol (Figs. 4A, 4B). Fifteen-minute treatment with 10 ng/ml TNF-α induced translocation of RelA to the nucleus, as shown by the uniform staining of both cytosol and nucleus (Fig. 4C). FHA pretreatment reduced the nuclear translocation of RelA induced by TNF-α (Fig. 4D). These findings support the proposed partial inhibition of IκBα proteasomal degradation by FHA, as previously discussed (Fig. 3A).

### Proteasomal activity is blunted by FHA treatment

**Figure 5:**
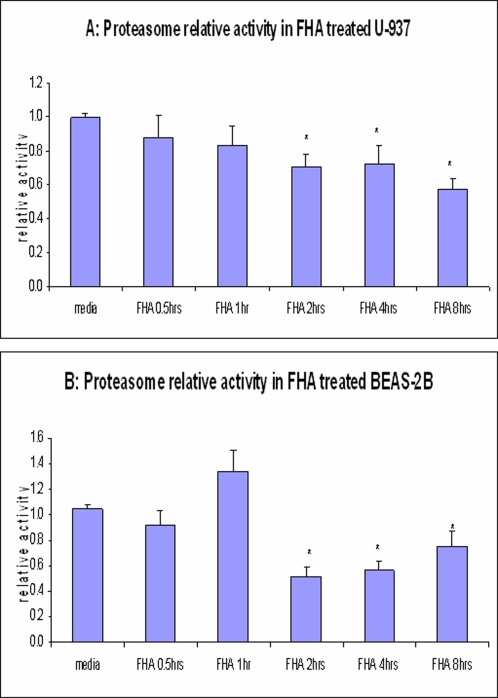
Proteasome activity in U-937 monocyte-derived macrophages and BEAS-2B bronchial epithelial cells. Proteasome activity was determined in lysates of U-937 monocyte-derived macrophages (A) and BEAS-2B bronchial epithelial cells (B) by incubating the lysates with the proteasome substrate Suc-LLVY-AMC, and then measuring the accumulation of free AMC fluorophore, which is cleaved from the substrate LLVY-AMC by the proteasome. These results are the mean ratios from six separate experiments, as described in Methods. Measurements were performed in quadruplicate and statistical significance was determined by the paired Student t-test. The reductions in proteasome activity were statistically significant (*) at 2 hr, 4 hr, and 8 hr (p<0.001) for both cell types.

Other data suggested that FHA does not interfere with the phosphorylation and ubiquitination of IκBα − functions that are needed for proteasomal degradation (Fig. S4) – we further examined whether prolonged treatment with FHA directly affects proteasome activity. Whole-cell lysates were prepared from cells treated with FHA for various periods of time (0–8 hours). The lysates were then incubated with fluorogenic proteasome-specific substrate, as described in Material and Methods. As illustrated in U-937 macrophages, FHA induced a 30, 27 and 43% inhibition of proteasomal activity at two, four and eight hours, respectively, in comparison to untreated macrophages (Fig. 5A). In BEAS-2B cells, FHA induced a 48, 44 and 25% inhibition at two, four and eight hours, respectively, in comparison to untreated cells (Fig. 5B). These results imply that longer exposures of both macrophages and epithelial cells to FHA result in attenuated proteasome function, which may contribute to reduced NF-κB pathway activity.

## Discussion

A well-described feature of mammalian innate responses to microorganisms involves recognition of common molecular patterns, such as lipopolysaccharide and peptidoglycan, and subsequent activation of the NF-κB transcription factor pathway. It is not surprising that some pathogens, such as *Y. pestis*, *Y. enterocolitica* and *Salmonella enterica* Typhimurium, have evolved mechanisms for exploiting this regulatory system as a means for securing a host niche [16]–[18]. Among the bordetellae, a type III-secretion system in *B. bronchiseptica* is responsible for inhibition of the NF-κB pathway in murine macrophages [19], [20].

Virulence factors act in concert during natural infections of *B. pertussis*. Under these circumstances, it is difficult or impossible to dissect out the contributions of individual factors. FHA is well characterized as an adhesin. However, the significant amounts of FHA secreted by *B. pertussis* have led to speculation that this soluble molecule has an additional role in pathogenesis. Although the amounts or concentrations of secreted FHA *in vivo* are unclear, we see great value in understanding the potential consequences of FHA interactions with host cells. We focused on the NF-κB response system because of its central role in inflammation and because of previous observations of FHA-associated TNF-α secretion and apoptosis by mononuclear leukocytes [8]. We measured NF-κB activity in two cell types, both of which are relevant to *B. pertussis* infection: mononuclear leukocytes, i.e., a monocyte-derived macrophage cell-line (U-937) and fresh human monocytes. We also analyzed the interactions of FHA with a cell-line model of bronchial epithelial cells, which is a major target cell type for *B. pertussis.*

Our results revealed a dynamic, time-dependent NF-κB pathway response to FHA where, despite the initial rapid activation of the pathway in macrophages, prolonged exposure to FHA inhibited this pathway and suppressed subsequent responses to other inflammatory signals. In contrast, in bronchial epithelial cells, FHA blocked activation of the NF-κB pathway at all examined time points. Cells were analyzed with various assays that assess the activation state of the NF-κB pathway, including IκBα degradation, nuclear translocation of the transcription factor, DNA binding of the transcription factor, and the secretion of NF-κB-regulated cytokines, such as TNF-α and IL-8. Up-regulated IL-6 and TNF-α secretion were previously reported in the murine macrophage cell line J774 following treatment with soluble FHA [7]. The chemokine IL-8 was detected in the supernatant of epithelial cells exposed to FHA; however, since NF-κB is not activated by FHA in these cells, we speculate that a different transcription factor is responsible for IL-8 transcription. NF-κB is believed to be expressed in BEAS-2B epithelial cells, based on previous microarray-derived data [21] and based on the NF-κB pathway-associated responses of BEAS-2B cells to TNF-α observed in this study.

FHA is purified from liquid cultures of *B. pertussis*. LPS levels were previously measured in the FHA samples and found to be very low but not absent [8]. However, we previously showed that *B. pertussis* LPS is a weak inducer of inflammatory responses, as well as apoptosis, in the same cell types, when compared to FHA [8]. To verify if the initial inflammatory response might be due in part to the minute amounts of contaminating LPS, we pretreated FHA with 10 µg/ml polymixin B and then used this preparation to treat differentiated U-937 cells. We found that IκBα was degraded by 0.5 hour following exposure to either polymixin B-pretreated FHA or untreated FHA, suggesting that LPS does not contribute significantly to the rapid activation of NF-κB by FHA (Fig. S5).

It was previously suggested that FHA binds to macrophages through α_M_β_2_ integrin receptors [5]. These receptors are capable of activating NF-κB [22]. However, it remains to be determined whether this is the mechanism by which FHA activates NF-κB. It is possible that the different responses of macrophages and epithelial cells originate in the distinct repertoire of receptors expressed by these cells. A prior study showed that in BEAS-2B cells exposed to FHA-coated wells, the NF-κB pathway is activated in an RGD motif-dependent manner [23]. We speculate that differences in the presentation of FHA (surface-associated versus soluble) may account for the discrepancy in the responses identified in BEAS-2B epithelial cells. Since cells are exposed *in vivo* to both surface-associated FHA and soluble FHA, both models of FHA presentation are relevant.

As reported by others, we observed high levels of secretion of the anti-inflammatory cytokine IL-10 by U-937 macrophages and fresh human monocytes exposed to FHA. Additional immunosuppressive activities have been attributed to FHA, and associated with the induction of T regulatory cells. IL-10 was shown to disrupt NF-κB activation by interfering with IκBα degradation [24] and IKKβ−induced phosphorylation [25]. Further analyses are needed to determine whether IL-10 alone mediates FHA inhibitory effects in macrophages. However, IL-10 is not secreted by epithelial cells and thus cannot explain the inhibitory effects in BEAS-2B cells.

The inhibition of proteasomal activity in cells treated for prolonged periods of time with FHA in this study may explain the limited degradation of IκBα in these cells and may offer support for our previous published observations, where similar conditions induced cell apoptosis [8]. The accumulation of IκBα has been shown in other systems to lead to apoptosis [26]. Other pathogens produce factors that are proteasome inhibitors. In fact, one of the most popular proteasome inhibitors used in laboratory experiments is lactacystin, which is produced by *Streptomyces* [27]. Interestingly, proteasome inhibition was previously shown to increase IL-10 secretion by the promonocytic cell line THP-1 in response to LPS [28]. The ability of FHA to interfere with such a critical function as proteasome activity may explain its role in the attenuation of the immune response to *Bordetella* infection. Our data suggest a previously unrecognized mechanism of action for the soluble-secreted form of FHA. Given that FHA is a component of acellular pertussis vaccines, further examination of these activities may be prudent.

## Supporting Information

Figure S1 Specificity and super-shift analysis of NF-κB DNA binding activity in U-937 macrophages. Nuclear extracts from U-937 derived macrophages treated with media for 8 hours, 5 µg/ml FHA for 8 hours, or TNF-α for 15 min were incubated for one hour with or without unlabeled NF-κB oligo or with anti-p50 antibody as described and analyzed for DNA binding by EMSA. Incubation with anti-p50 antibody resulted in a shift or delay of the p50 subunit migration, as indicated in the figure, confirming its contribution to the RelA/p50 transcription factor band. This is representative of three assays performed under similar conditions.
(0.04 MB TIF)

Figure S2 TNF-α and LPS reveal proteosome-dependent NF-κB activation that is attenuated by FHA. Fresh human monocytes were pre-treated as indicated with media or 10 µM ALLN208719 or 5 µg/ml FHA and then activated for 15 minutes with 10 ng/ml TNF-α or 60 minutes with 10 µg/ml *Bordetella pertussis* LPS. Cytoplasmic extracts were analyzed by immunoblot procedures with an antibody directed against the C-terminus of IκBα. This is a representative experiment of three performed under similar conditions.
(0.07 MB TIF)

Figure S3 FHA attenuates NFκB activity triggered by TNF-α, LPS or IL-1β in human monocytes and BEAS-2B cells. Fresh human monocytes (A) or BEAS-2B cells (B) were pre-treated as indicated, with media or 5 µg/ml FHA and then exposed for 15 minutes to 10 ng/ml TNF-α or 60 minutes with 10 µg/ml *Bordetella pertussis* LPS or 60 minutes with 50 ng/ml IL-1β, as indicated. Cytoplasmic extract protein was analyzed with immunoblot procedures using an antibody directed against the C-terminus of IκBα. This is a representative experiment of three performed under similar conditions.
(0.08 MB TIF)

Figure S4 Phosphorylation and ubiquitination of IκBα in BEAS-2B and U-937 cells induced by FHA. A: BEAS-2B cells and B: differentiated U-937 cells, as described in methods, were pretreated with 10 µM ALLN208719 and then with media alone, or 5 µg/ml FHA, or 10 ng/ml TNF-α, or 5 µg/ml FHA and then 10 ng/ml TNF-α, for the times indicated. Equal amounts of whole cell lysate were immunoprecipitated with anti-IκBα antibody and immunoblotted with anti-ubiquitinin or p-ser-IκBα as indicated. This is a representative assay out of three assays performed under similar conditions.
(0.08 MB TIF)

Figure S5 IκBα cytosolic levels in FHA-treated U-937 cells: Pretreatment with polymixin B, MG-132 or calpain inhibitors. U-937 cells were pretreated for 60 minutes with 10 µM MG-132 or 10 µM calpain and then incubated with 5 µg/ml FHA for the times indicated. Additionally, cells were treated for the times indicated with 5 µg/ml FHA which had been pretreated for 60 min with 10 µg/ml polymixin B. Cytoplasmic extract protein was analyzed with immunoblot procedures using an antibody directed against the C-terminus of IκBα. This is a representative experiment of three performed under similar conditions.
(0.91 MB TIF)
