# Supplementary figures and images for: Modulation of the NF-κB Pathway by Bordetella pertussis Filamentous Hemagglutinin
Source: PLoS One. 2008 Nov 27;3(11):e3825. doi: 10.1371/journal.pone.0003825 (PMC2584786; doi:10.1371/journal.pone.0003825)

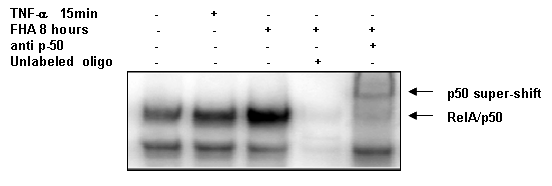

Supplement: Figure S1 — Specificity and super-shift analysis of NF-κB DNA binding activity in U-937 macrophages. Nuclear extracts from U-937 derived macrophages treated with media for 8 hours, 5 µg/ml FHA for 8 hours, or TNF-α for 15 min were incubated for one hour with or without unlabeled NF-κB oligo or with anti-p50 antibody as described and analyzed for DNA binding by EMSA. Incubation with anti-p50 antibody resulted in a shift or delay of the p50 subunit migration, as indicated in the figure, confirming its contribution to the RelA/p50 transcription factor band. This is representative of three assays performed under similar conditions. (0.04 MB TIF) [file pone.0003825.s001.tif]

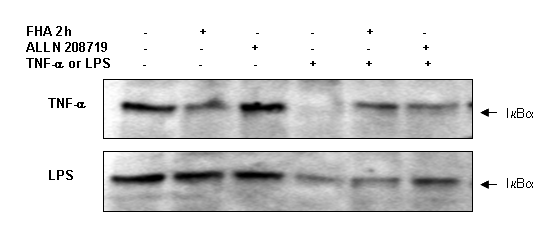

Supplement: Figure S2 — TNF-α and LPS reveal proteosome-dependent NF-κB activation that is attenuated by FHA. Fresh human monocytes were pre-treated as indicated with media or 10 µM ALLN208719 or 5 µg/ml FHA and then activated for 15 minutes with 10 ng/ml TNF-α or 60 minutes with 10 µg/ml Bordetella pertussis LPS. Cytoplasmic extracts were analyzed by immunoblot procedures with an antibody directed against the C-terminus of IκBα. This is a representative experiment of three performed under similar conditions. (0.07 MB TIF) [file pone.0003825.s002.tif]

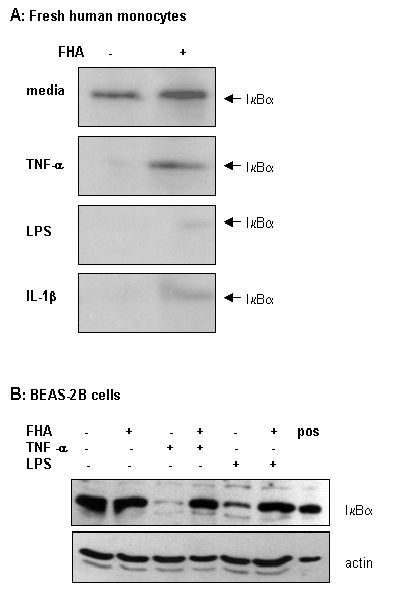

Supplement: Figure S3 — FHA attenuates NFκB activity triggered by TNF-α, LPS or IL-1β in human monocytes and BEAS-2B cells. Fresh human monocytes (A) or BEAS-2B cells (B) were pre-treated as indicated, with media or 5 µg/ml FHA and then exposed for 15 minutes to 10 ng/ml TNF-α or 60 minutes with 10 µg/ml Bordetella pertussis LPS or 60 minutes with 50 ng/ml IL-1β, as indicated. Cytoplasmic extract protein was analyzed with immunoblot procedures using an antibody directed against the C-terminus of IκBα. This is a representative experiment of three performed under similar conditions. (0.08 MB TIF) [file pone.0003825.s003.tif]

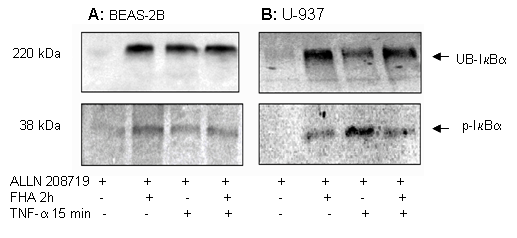

Supplement: Figure S4 — Phosphorylation and ubiquitination of IκBα in BEAS-2B and U-937 cells induced by FHA. A: BEAS-2B cells and B: differentiated U-937 cells, as described in methods, were pretreated with 10 µM ALLN208719 and then with media alone, or 5 µg/ml FHA, or 10 ng/ml TNF-α, or 5 µg/ml FHA and then 10 ng/ml TNF-α, for the times indicated. Equal amounts of whole cell lysate were immunoprecipitated with anti-IκBα antibody and immunoblotted with anti-ubiquitinin or p-ser-IκBα as indicated. This is a representative assay out of three assays performed under similar conditions. (0.08 MB TIF) [file pone.0003825.s004.tif]

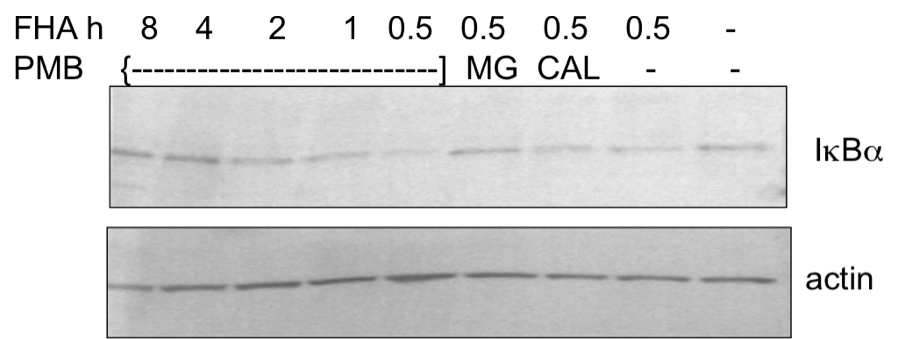

Supplement: Figure S5 — IκBα cytosolic levels in FHA-treated U-937 cells: Pretreatment with polymixin B, MG-132 or calpain inhibitors. U-937 cells were pretreated for 60 minutes with 10 µM MG-132 or 10 µM calpain and then incubated with 5 µg/ml FHA for the times indicated. Additionally, cells were treated for the times indicated with 5 µg/ml FHA which had been pretreated for 60 min with 10 µg/ml polymixin B. Cytoplasmic extract protein was analyzed with immunoblot procedures using an antibody directed against the C-terminus of IκBα. This is a representative experiment of three performed under similar conditions. (0.91 MB TIF) [file pone.0003825.s005.tif]
